# Liver metal levels and expression of genes related to iron homeostasis in rhesus monkeys after inhalational manganese exposure

**DOI:** 10.1016/j.dib.2016.01.055

**Published:** 2016-02-08

**Authors:** Michael A. Pettiglio, Carolina Herrera, Melanie L. Foster, David C. Dorman, Thomas B. Bartnikas

**Affiliations:** a Brown University, Department of Pathology and Laboratory Medicine, 70 Ship St., Rm. 522, Providence, RI 02912, USA; b North Carolina State University, College of Veterinary Medicine, 1060 William Moore Drive, Raleigh, NC 27607, USA

**Keywords:** Iron, Manganese, Inhalation, Monkey

## Abstract

Here we present data on liver metal levels and expression of genes related to iron homeostasis in rhesus monkeys after inhalational manganese exposure. Archived liver samples from rhesus monkeys exposed to 0 (*n*=6), 0.06 (*n*=6), 0.3 (*n*=4) and 1.5 (*n*=4) mg/m^3^ manganese inhalation for 65 days were obtained from a published study (“Tissue manganese concentrations in young male rhesus monkeys following subchronic manganese sulfate inhalation” [1]). Samples were analyzed by spectroscopy, immunoblotting and quantitative PCR to assess metal levels and gene expression. Liver manganese and iron levels were linearly correlated although only the intermediate manganese exposure level (0.3 mg Mn/m^3^) led to a statistically significant increase in liver iron levels.

**Specifications table**

**Table:** 

Subject area	*Toxicology*
More specific subject area	*Toxicology of metals*
Type of data	*Figures and tables*
How data was acquired	*Spectroscopy; immunoblotting; quantitative PCR (see materials and methods for instrument specifics)*
Data format	*Analyzed*
Experimental factors	*Archived liver samples from monkeys subjected to inhalational manganese exposure*
Experimental features	*Archived monkey liver samples were analyzed by spectroscopy, immunoblotting and quantitative PCR to assess metal levels and gene expression.*
Data source location	*Not applicable*
Data accessibility	*Data is within this article*

Value of the data

• Inhalational manganese exposure perturbed liver iron levels and expression of BMP6, a known regulator of systemic iron homeostasis.
• While sample size in this study is small, the potential value of this data is that it includes an analysis of factors involved in both cellular and systemic iron homeostasis.
• Our analysis may be useful as a reference for future, larger studies.

## 1 Data

**Fig. 1:**
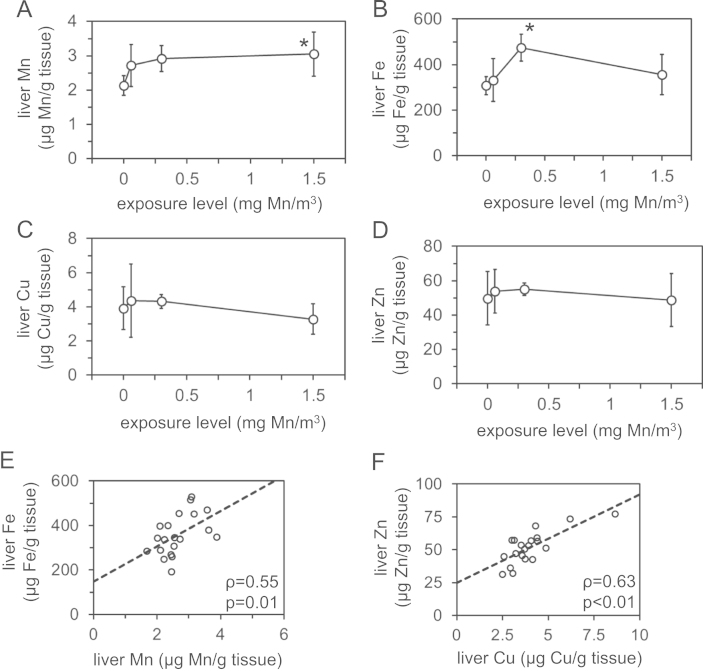
Liver metal levels in rhesus monkeys after manganese inhalation**.** (A–D) Levels of manganese (Mn, A), iron (Fe, B), copper (Cu, C) and zinc (Zn, D) were measured by ICP-AES and GF-AAS in livers from monkeys subjected to 0–1.5 mg/m^3^ manganese inhalation for 65 days. Circles represent average values from 4–6 livers; bars indicate standard deviation. ^⁎^ Indicates statistical significance (*p*<0.05) compared to control group (0 mg/m^3^) as calculated by one-way ANOVA with Dunnett׳s method. (E and F) Liver Mn vs. Fe levels (E) and Cu vs. Zn levels (F) were plotted along with rho (*ρ*) and *p* values as calculated by Spearman׳s rank correlation test.

**Fig. 2:**
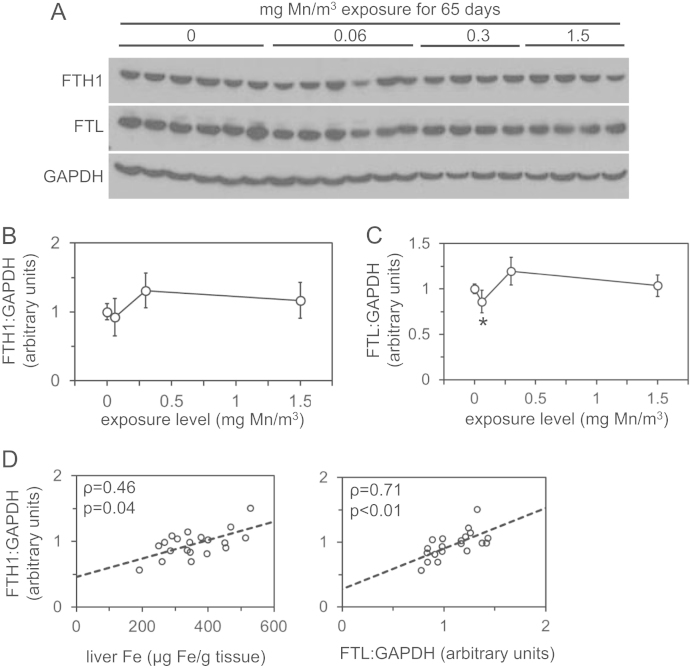
Liver ferritin levels in rhesus monkeys after manganese inhalation**.** (A) Levels of ferritin heavy chain (FTH1) and light chain (FTL) and GAPDH were measured by immunoblot in protein lysates extracted from livers of monkeys after manganese inhalation. (B and C) Levels of immunoblot signal for FTH1 (B) and FTL (C) were measured relative to GAPDH signal by densitometry and plotted vs. exposure level. ^⁎^ Indicates statistical significance (*p*<0.05) compared to control group (0 mg/m^3^) as calculated by one-way ANOVA with Dunnett׳s method. (D) Liver FTH1:GAPDH ratios were plotted against liver iron (Fe) levels and FTL:GAPDH levels along with rho (*ρ*) and *p* values as calculated by Spearman׳s rank correlation test.

**Fig. 3:**
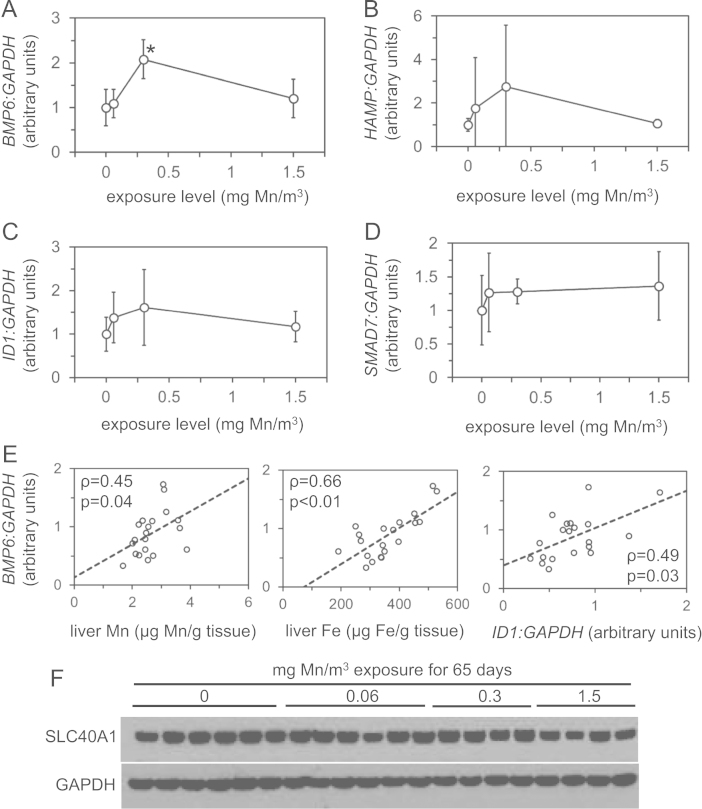
Expression levels of factors in the BMP6-HAMP-SLC40A1 iron trafficking axis in livers from rhesus monkeys after manganese inhalation. (A–D) Liver RNA levels of bone morphogenetic protein 6 (*BMP6),* hepcidin antimicrobial peptide (*HAMP*), inhibitor of differentiation 1 (*ID1)* and mothers against decapentaplegic homology 7 (*SMAD7)* were measured by QPCR and plotted relative to liver *GAPDH* RNA levels vs. manganese exposure level. ^⁎^ Indicates statistical significance (*p*<0.05) compared to control group (0 mg/m^3^) as calculated by one-way ANOVA with Dunnett׳s method. (E) Liver *BMP6:GAPDH* ratios were plotted vs. liver manganese (Mn), iron (Fe) and *ID1:GAPDH* levels along with rho (*ρ*) and *p* values as calculated by Spearman׳s rank correlation test. (F) Levels of SLC40A1 (ferroportin) were measured by immunoblot in protein lysates extracted from livers of monkeys after manganese inhalation.

**Fig. 4:**
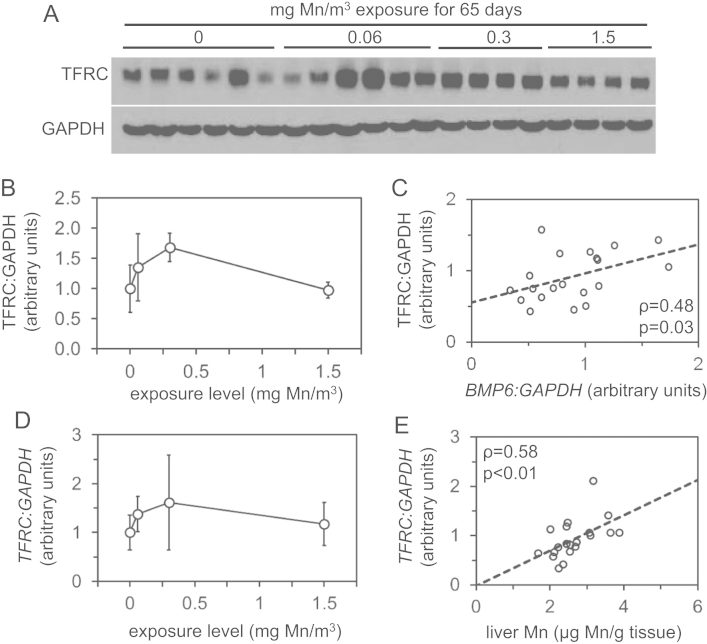
Liver transferrin receptor levels in rhesus monkeys after manganese inhalation**.** (A) Levels of transferrin receptor (TFRC) and GAPDH were measured by immunoblot in protein lysates extracted from livers of monkeys after manganese inhalation. (B) Liver TFRC:GAPDH ratios were plotted relative to manganese (Mn) exposure level. (C) Liver TFRC:GAPDH ratios were plotted vs. *BMP6:GAPDH* RNA levels along with rho (*ρ*) and *p* values as calculated by Spearman׳s rank correlation test. (D) Liver *TFRC* and *GAPDH* RNA levels were measured by QPCR and plotted as a ratio vs. manganese exposure level. (E) Liver *TFRC:GAPDH* ratios were plotted relative to liver manganese (Mn) levels along with rho (*ρ*) and *p* values as calculated by Spearman׳s rank correlation test.

**Fig. 5:**
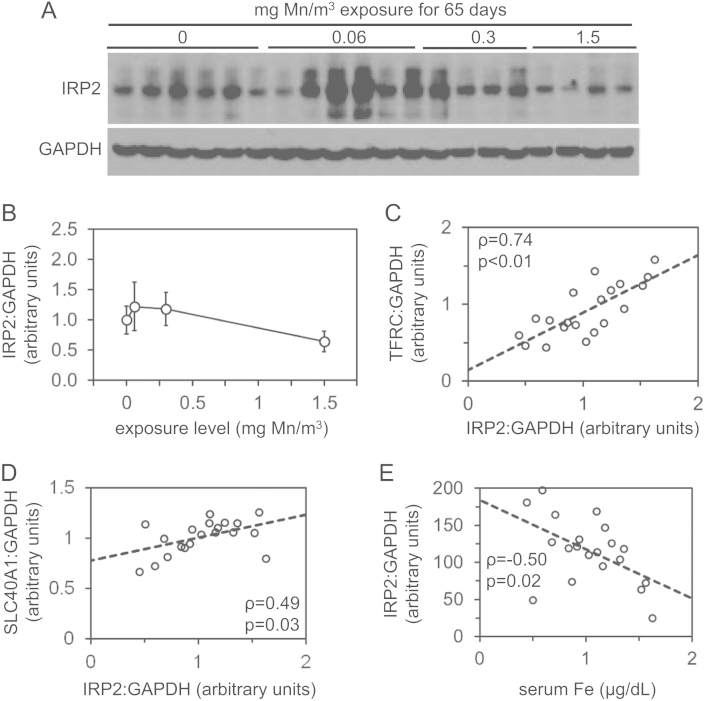
Liver iron regulatory protein 2 levels in rhesus monkeys after manganese inhalation**.** (A) Levels of iron regulatory protein 2 (IRP2) and GAPDH were measured by immunoblot in protein lysates extracted from livers of monkeys after manganese inhalation. (B) Liver IRP2:GAPDH ratios were plotted relative to manganese (Mn) exposure level. (C–E) Liver TFRC:GAPDH ratios (C), SLC40A1:GAPDH ratios (D) and serum iron (Fe) levels were plotted vs. IRP2:GAPDH levels. rho (*ρ*) and *p* values as calculated by Spearman׳s rank correlation test are indicated.

We analyzed levels of manganese, iron, copper and zinc and expression of genes involved in iron homeostasis in archived liver samples from rhesus monkeys after inhalational manganese exposure [1]. Fig. 1 depicts liver levels of manganese, iron, copper and zinc. Fig. 2, Fig. 3, Fig. 4, Fig. 5 depict levels of various factors involved in iron storage, export, import and regulation of gene expression. Specifically, Fig. 2 depicts levels of ferritin, an intracellular iron storage protein abundantly expressed in conditions of iron excess. Fig. 3 depicts levels of factors that regulate cellular iron excess: BMP6, a protein abundantly expressed in conditions of iron excess; HAMP, a liver-derived hormone also known as hepcidin that negatively regulates iron excess and is stimulated by BMP6; SLC40A1, a cellular iron export protein also known as ferroportin that is negatively regulated by HAMP. Fig. 4 depicts levels of transferrin receptor, an iron import protein that mediates cellular uptake of iron bound to the serum protein transferrin. Fig. 5 depicts levels of iron regulatory protein 2, an RNA-binding protein that regulates expression of genes such as ferritin and transferrin receptor in response to changes in cellular iron levels. Table 1 presents analysis of correlations between all measured parameters in Fig. 1, Fig. 2, Fig. 3, Fig. 4, Fig. 5. Table 2 presents hematologic data previously referenced as data not shown in the original study [1].

## 2 Experimental design, materials and methods

### 2.1 Animals

Archived samples of rhesus monkey liver were obtained from a previously published study [1]. Details concerning the animals, their husbandry and manganese inhalation have been published [1]. Male rhesus monkeys purchased from Covance Research Products, Inc. (Alice, TX) were used. Monkeys were approximately 20–24 months old at the start of the inhalation exposure. Monkeys were exposed to MnSO_4_ for 6 h/day, 5 days/week, for 13 weeks (65 exposure days). These monkeys were allocated as follows: 0.0 (*n*=6), 0.06 (*n*=6), 0.3 (*n*=4), and 1.5 (*n*=4) mg Mn/m^3^. Food was withheld overnight prior to necropsy. Monkeys were anesthetized with ketamine (20 mg/kg, i.m., Fort Dodge Animal Health, Fort Dodge, IA), blood collected, and then euthanized with pentobarbital (80–150 mg/kg, i.v., Henry Schein, Inc., Port Washington, NY) followed by exsanguination. Liver samples were stored in individual plastic vials or bags, frozen in liquid nitrogen, and stored at approximately −80 °C until analyses were performed.

### 2.2 Tissue analyses

Frozen liver samples (50–100 mg) were thawed and digested in 1 mL trace metal grade nitric acid (Fisher) at 65 °C for 2 h, then diluted 25-fold with MilliQ-purified water (Millipore). Digested samples were analyzed for manganese and iron concentrations in triplicate by inductively coupled plasma atomic emission spectrometry (ICP-AES) using a JY2000 Ultrace spectrometer (Horiba) or by graphite furnace atomic absorption spectrometry (GF-AAS) using an AAnalyst 600 spectrometer (Perkin Elmer) in the Environmental Chemistry Facility at Brown University using previously described protocols [2]. Reference standards were analyzed repeatedly during each run to ensure run consistency. Additional samples of liver were used to assess biochemical endpoints. RNA was extracted from thawed liver tissue and analyzed by quantitative polymerase chain reaction (QPCR) using Taqman assays (Life Technologies) Rh02839540_m1 (*BMP6*), Rh02819165_m1 (*HAMP*), Rh02913303_m1 (*ID1*), Rh00998191_m1 (*SMAD7*), Rh02621719_u1 (*IL6*), Rh02621758_m1 (*TFRC*) and Rh02621745_g1 (*GAPDH*) as previously described [2]. Proteins were extracted from liver tissue and immunoblotted as previously described [2] using antibodies against ferritin heavy chain (FTH1) (Cell Signaling), ferritin light chain (FTL) (Proteintech), glyceraldehyde 3-phosphate dehydrogenase (GAPDH) (Cell Signaling), ferroportin/SLC40A1 (Novus), transferrin receptor (TFRC) (Proteintech) and iron regulatory protein 2 (IRP2) (Novus). Densitometry of immunoblots was performed using ImageJ [3].

### 2.3 Statistics

Following an assessment of normality by Shapiro–Wilk test, the data for continuous variables were inter-compared for the exposure groups by analysis of variance (ANOVA). If the exposure`s main effect was significant, a Dunnett׳s test was used to compare the three MnSO_4_ exposure levels to the air-exposed controls. Unless otherwise noted, data presented are for the mean values±standard deviation. Correlations between measured parameters were assessed by Spearman Rank Order Correlation test. A probability value of *p*=0.05 was used as the critical level of significance for all statistical tests. Statistical analysis was performed using Sigmaplot (Systat Software). Analysis did not take into account effect of multiple comparisons on calculation of statistical significance.

## Figures and Tables

**Table 1:** Spearman׳s rank correlations between parameters of iron homeostasis in monkeys exposed to inhalation of 0–1.5 mg Mn/m^3^ over 65 days.

*ρ* and *p* values are indicated on the first and second lines of each box respectively. Boxes are shaded gray if *p*<0.05 Parameters include: liver manganese (Mn), iron (Fe), copper (Cu) and zinc (Zn) levels (µg/g liver), liver RNA and protein levels (relative to GAPDH RNA and protein levels respectively; arbitrary units), plasma Fe levels (µg/dL), plasma transferrin (TF) levels (mg/dL), total iron binding capacity (TIBC; mg/dL), TF saturation (Tf sat; %), red blood cell count (RBC; ×10^6^/µL), hemoglobin (HGB; g/dL), hematocrit (HCT; %), mean corpuscular volume (MCV; fL), mean corpuscular hemoglobin (MCH; pg); mean corpuscular hemoglobin concentration (MCHC; %).

**Table 2:** Hematological parameters in monkeys exposed to inhalation of 0–1.5 mg Mn/m^3^ over 65 days.

	Manganese Exposure Concentration (mg Mn/m^3^)			
Parameter	0	0.06	0.3	1.5
Fe	115.5±25.5	91.7±54.7	100.5±22.0	165.3±33.7
TF	208.8±18.7	223.0±27.2	210.8±23.1	222.3±34.4
TIBC	331.8±44.1	356.8±55.9	344.3±45.9	363.8±53.2
TF sat	34.8±6.2	25.9±15.0	29.2±5.6	46.9±14.5
RBC	4.9±0.3	5.1±0.4	4.9±0.2	5.3±0.3
HGB	12.8±0.5	12.4±0.6	12.2±0.8	13.4±0.8
HCT	36.5±1.5	35.8±1.4	34.6±2.2	38.7±2.5
MCV	74.5±2.7	71.5±5.1	71.5±1.3	73.0±1.8
MCH	26.1±1.2	24.7±2.2	25.1±0.5	25.3±0.7
MCHC	35.1±0.4	34.6±0.6	35.2±0.1	34.6±0.6

In each box, the average value±standard deviation is shown. Parameters include: plasma iron (Fe) levels (µg/dL), plasma transferrin (TF) levels (mg/dL), total iron binding capacity (TIBC; mg/dL), TF saturation (Tf sat; %), red blood cell count (RBC; ×10^6^/µL), hemoglobin (HGB; g/dL), hematocrit (HCT; %), mean corpuscular volume (MCV; fL), mean corpuscular hemoglobin (MCH; pg); mean corpuscular hemoglobin concentration (MCHC; %). None of the 0.06, 0.3 and 1.5 mg Mn/m^3^ values differed significantly (*p*<0.05) from control (0 mg Mn/m^3^) as calculated by one-way ANOVA. These values were collected during the original study [1] and are shown here for completeness.
