# Supplementary material for: Liver metal levels and expression of genes related to iron homeostasis in rhesus monkeys after inhalational manganese exposure
Source: Data Brief. 2016 Feb 8;6:989–97. doi: 10.1016/j.dib.2016.01.055 (PMC4763106; doi:10.1016/j.dib.2016.01.055)
Supplement: Supplementary file 1 — Supplementary material [file mmc1.pdf]

BROWN
Alpert Medical School

Department of Pathology
& Laboratory Medicine

December 30, 2015

Dear Editors,

I am writing this letter to confirm that there are no known conflicts of interest associated with this publication and there has been no significant financial support for this work that could have influenced its outcome.

Sincerely,

Tom Bartnikas

(thomas_bartnikas@brown.edu)

Brown University Warren Alpert Medical School Box G-E5, Providence, RI 02912 TEL: 401 863-3119 FAX: 401 863-9008
